# The Anatomy and Operative Surgery of Inguinal and Femoral Hernia

**Published:** 1834-07-01

**Authors:** 


*The Anatomy and Operative Surgery of Inguinal and Femoral Hernia.* By VALENTINE FLOOD, A.M., M.D., &c. *Illustrated by eight Folio Plates.*—London, 1834.

THESE plates are of neat, but by no means of brilliant execution: they are however clear and distinct, and will serve the purposes of the student. It is with a feeling akin to regret we perceive that the violent spirit of competition, which is the canker of our profession,—which makes gratuitous offices sought for with breathless eagerness, and threatens to reduce medical remuneration to the level of the wages of a day-labourer, seems to pervade even medical literature. Mr. Guthrie, Mr. Bloxam, Mr. Tuson, and Dr. Flood, have all, within a few months, published plates intended to lessen the difficulties of the operation for strangulated hernia; yet the author says "there is still a great deficiency of plates illustrating operative surgery."

Dr. Flood intends to illustrate all the capital operations in surgery in a similar manner; so does Mr. Bloxam, to say nothing of Bourgery. We trust that they may all prosper; but they must recollect that, when so many rivals are in the field, nothing but the most finished excellence can obtain attention, far less success.

